# Supplementary material for: Anatomical Characteristics of Cervicomedullary Compression on MRI Scans in Children with Achondroplasia
Source: J Imaging. 2024 Nov 14;10(11):291. doi: 10.3390/jimaging10110291 (PMC11595703; doi:10.3390/jimaging10110291)
Supplement: Supplementary file 1 [file jimaging-10-00291-s001.zip › jimaging-3264353-supplementary.pdf]

Table S1. Analysis of anatomical parameters on magnetic resonance imaging scans in children with achondroplasia compared with the reference group.

Parameter		Achondroplasia	Reference	*p*-Value *
Foramen magnum diameter, mm	No. patients	33	33	< 0.001
	Mean (SD)	10.0 (2.3)	16.1 (3.4)	
	Median (range)	9.5 (5.5–16.6)	15.0 (8.0–23.5)	
Foramen magnum area, mm²	No. patients	33	33	0.005
	Mean (SD)	109.0 (31.5)	160.8 (76.6)	
	Median (range)	115.0 (49.6–163.8)	151.6 (64.3–389.1)	
Myelon area, mm²	No. patients	33	34	0.004
	Mean (SD)	40.6 (16.1)	47.7 (10.2)	
	Median (range)	37.0 (16.5–102.3)	47.6 (31.4–81.9)	
Clivus length, mm	No. patients	36	36	< 0.001
	Mean (SD)	23.5 (3.1)	30.3 (3.5)	
	Median (range)	23.0 (16.0–32.5)	30.7 (23.3–36.0)	
Tentorium angle, degrees	No. patients	35	37	< 0.001
	Mean (SD)	47.6 (5.5)	38.1 (4.7)	
	Median (range)	47.5 (31.5–58.0)	37.0 (27.5–50.0)	
Occipital angle, degrees	No. patients	35	37	0.005
	Mean (SD)	77.9 (6.6)	83.2 (6.5)	
	Median (range)	79.0 (64.0–89.5)	82.0 (72.0–99.0)	
Posterior fossa volume, mm³	No. patients	24	37	0.836
	Mean (SD)	126,080.6 (30,947.6)	121,382.3 (42,399.2)	
	Median (range)	126,330.3 (48,439.7–177,870.0)	138,058.6 (30,111.9–196,052.3)	
Proportion of brain-stem volume outside the posterior fossa, mm³	No. patients	24	36	< 0.001
	Mean (SD)	4542.5 (1089.9)	2614.4 (868.0)	
	Median (range)	4448.4 (2778.6–7021.3)	2746.8 (760.3–4016.8)	
Cerebellum volume, mm³	No. patients	24	37	0.118
	Mean (SD)	74,887.3 (45,109.0)	89,842.0 (33,944.2)	
	Median (range)	85,526.0 (98.7–141,281.0)	104,499.4 (20,466.3–133,517.3)	
Supratentorial ventricular system volume, mm³	No. patients	32	34	< 0.001
	Mean (SD)	42,062.3 (24,302.4)	13,389.2 (4439.0)	
	Median (range)	35,651.9 (10,782.3–115,394.5)	13,828.9 (2434.7–24,621.7)	
Intracranial CSF system volume, mm³	No. patients	31	34	< 0.001
	Mean (SD)	225,804.8 (76,082.3)	126,716.7 (62,550.4)	
	Median (range)	206,853.0 (118,999.2–376,599.0)	113,004.7 (9470.5–298,628.8)	
Fourth ventricle volume, mm³	No. patients	35	37	0.029
	Mean (SD)	749.9 (355.3)	1056.2 (589.5)	
	Median (range)	667.4 (292.0–1607.8)	991.8 (224.6–2452.6)	

* Mann–Whitney U test for independent samples. CSF, cerebrospinal fluid; SD, standard deviation.
